# Clinical and CT features of mild-to-moderate COVID-19 cases after two sequential negative nucleic acid testing results: a retrospective analysis

**DOI:** 10.1186/s12879-021-06013-x

**Published:** 2021-04-08

**Authors:** Yan Rong, Fei Wang, Jinfei Tian, Xinhua Liang, Jing Wang, Xiaoli Li, Dandan Zhang, Jing Liu, Huadong Zeng, Yang Zhou, Yi Shi

**Affiliations:** 1 Department of Respiratory and Critical Care Medicine, Shenzhen Hospital, Southern Medical University, NO 1333, Xinhu Road, Baoan District, Shenzhen, 518100 China; 2 Department of Orthopaedics, The University of Hong Kong - Shenzhen Hospital, Shenzhen, 518053 China; 3 Department of Intensive Care Unit, Shenzhen Hospital, Southern Medical University, Shenzhen, 518100 China; 4 Department of Nephrology, Shenzhen Hospital, Southern Medical University, Shenzhen, 518100 China; 5 Department of Radiology, Shenzhen Hospital, Southern Medical University, Shenzhen, 518100 China; 6 Department of Health Management Center, Shenzhen Hospital, Southern Medical University, Shenzhen, 518100 China; 7 Department of Respiratory and Critical Care Medicine, Jinling Hospital, The First School of Clinical Medicine, Southern Medical University, Nanjing, 210002 China

**Keywords:** COVID-19, SARS-CoV-2, Computed tomography, Negative nucleic acid testing

## Abstract

**Background:**

The clinical and imaging features of patients with severe acute respiratory syndrome coronavirus 2 (SARS-CoV-2) infections that progressed to coronavirus disease 2019 (COVID-19) have been explored in numerous studies. However, little is known about these features in patients who received negative respiratory nucleic acid test results after the infections resolved. In this study, we aim to describe these features in a group of Chinese patients.

**Methods:**

This retrospective study includes 51 patients with mild-to-moderate COVID-19 (median age: 34.0 years and 47.1% male) between January 31 and February 28, 2020. Demographic, clinical, laboratory, and computed tomography (CT) imaging data were collected before and after two consecutive negative respiratory SARS-CoV-2 tests.

**Results:**

Following a negative test result, the patients’ clinical symptoms continued to recover, but abnormal imaging findings were observed in all moderate cases. Specifically, 77.4% of patients with moderate COVID-19 exhibited multi-lobar lung involvement and lesions were more frequently observed in the lower lobes. The most common CT imaging manifestations were ground-glass opacities (51.6%) and fibrous stripes (54.8%%). Twelve of the 31 patients with moderate COVID-19 underwent repeated chest CT scans after a negative SARS-CoV-2 test. Among them, the ground-glass opacities decreased by > 60% within 1 week in seven patients (58.3%), but by < 5% in four patients (13.8%).

**Conclusions:**

Following a positive and subsequent negative SARS-CoV-2 tests, patients with COVID-19 continued to recover despite exhibiting persistent clinical symptoms and abnormal imaging findings.

**Supplementary Information:**

The online version contains supplementary material available at 10.1186/s12879-021-06013-x.

## Introduction

In December 2019, cases of “pneumonia of unknown cause” were first reported in Wuhan city, Hubei province, China. Subsequently, the novel causative pathogen of this emerging outbreak, severe acute respiratory syndrome coronavirus 2 (SARS-CoV-2), was first isolated on January 7, 2020 [1]. On January 30, 2020, the International Health Regulations Emergency Committee of the World Health Organization (WHO) declared coronavirus disease 2019 (COVID-19) to be a Public Health Emergency of International Concern (PHEIC) [2]. As of March 18, 2021, 120,383,919 cases of COVID-19 and 2,664,386 deaths have been confirmed by 223 countries, areas or territories worldwide [2]. Currently, the treatment and control of COVID-19 are still unknown and constitute a major public health issue [3].

Several large studies and meta-analyses have broadly described and summarized the clinical and imaging features of patients with COVID-19 to enable diagnosis in the early stage and assess the disease evolution [4–12]. Recently, one study conducted by Giannitto C et al. [13] reported that the negative result of reverse-transcription-polymerase chain-reaction (RT-PCR) test but the positive result of computed tomography (CT) indicated high risk of COVID-19 in a cluster or community transmission scenarios. Chen Z et al. [14] consistently found that clinical and CT imaging features might be different between patients with initial negative and later positive RT-PCR test of COVID-19. Another study consistently called for regular follow-up of CT scan because pulmonary lesions persisted among patients with COVID-19 even after twice consecutive negative nucleic acid test results [15]. However, there are still few studies focused on the progress of these features in patients with COVID-19 after they had received a negative SARS-CoV-2 test result. Milestone considered important in the context of slowing widespread community transmission and in decisions regarding isolation and hospital discharge.

The criteria for discharge after treatment for COVID-19 vary among countries and regions and depend on factors such as the existing healthcare capacity, laboratory diagnostic resources, and current epidemiological situation (https://www.ecdc.europa.eu/sites/default/files/documents/covid-19-guidance-discharge-and-ending-isolation-first%20update.pdf). In China, patients who are afebrile for > 3 days with improved respiratory symptoms, exhibiting obvious resolution of inflammation on pulmonary imaging, and receiving two consecutive negative respiratory SARS-CoV-2 PCR-based nucleic acid tests at an interval of ≥24 h, can be discharged from the hospital (http://www.nhc.gov.cn/). However, Wu et al. [16] reported that patients with negative respiratory tract sample results may still yield virus-positive fecal samples and the disease evolution after discharge remains unclear. No evidence-based practical measures have been developed to target this population, and therefore, the clinical characteristics of patients after negative nucleic acid testing are needed to provide guidance regarding these patients.

With this retrospective study, we aimed to describe the clinical and imaging characteristics of patients in Shenzhen, China, who had received two consecutive negative nucleic acid test results after a positive test-based COVID-19 diagnosis. Our results may provide evidence for the treatment of recovering COVID-19 patients and the prevention of virus transmission.

## Methods

### Study design and participants

This retrospective single-center study was conducted by using the data of all consecutive patients with confirmed COVID-19 who had been diagnosed at government-assigned hospitals in Shenzhen according to the Chinese Program for the Diagnosis and Treatment of COVID-19 [17] and were transferred to the Hezheng ward of Shenzhen Hospital, Southern Medical University, Shenzhen, China, between January 31 and February 28, 2020. All patients with confirmed COVID-19 were required to undergo daily SARS-CoV-2 nucleic acid testing until two consecutive negative results were obtained. Based on their overall condition, the patients were then discharged or transferred to other government-assigned hospitals for further treatment. The Hezheng ward was responsible for providing further treatment to patients who had obtained two consecutive negative results. The patients’ clinical characteristics and outcomes were monitored up to March 7, 2020, the last date of follow-up.

This study was approved by the Ethics Committee of Shenzhen Hospital, Southern Medical University.

### Data collection

Using structured data collection forms, the clinical characteristics and outcomes of the enrolled patients at the first admission due to COVID-19 and after transfer to the Hezheng ward after two consecutive negative nucleic acid test results were obtained from the electronic medical records. All data were reviewed by a trained team of physicians. The recorded information included demographic data, the epidemiological history, underlying comorbidities (e.g., bronchial or lung diseases and other comorbidities), symptoms, signs, laboratory parameters (e.g., white blood cell and lymphocyte counts, neutrophil and lymphocyte percentages, erythrocyte sedimentation rate, and C-reactive protein, D-dimer, lactate dehydrogenase, alanine aminotransferase, and aspartate aminotransferase concentrations), and chest CT scan data, if available. The patients’ treatment and outcome data were also extracted.

### Diagnosis of COVID-19 and clinical classification

COVID-19 cases were diagnosed according to the guideline for the prevention and control of COVID-19 (7th edition) published by General Office of National Health Committee of China [17]. An epidemiological history was defined as: 1) travelling to or residence in Wuhan or other cities with continuous and local SARS-CoV-2 transmission in the 14 days before symptom onset; 2) contacting with SARS-CoV-2-infected patients in the 14 days before symptom onset; 3) contacting with patients with a fever or respiratory symptom from Wuhan or other cities with continuous local transmission in the 14 days before symptom onset; or 4) clustered onsets. A suspected case was defined as a patient with an epidemiological history and any two clinical features of COVID-19 or without an epidemiological history but with all of the following clinical features: 1) fever and/or respiratory symptoms; 2) imaging features consistent with COVID-19; and 3) a normal or reduced total white blood cell count and/or normal or reduced lymphocyte count during the early stage of the disease. A confirmed case was further defined using the criteria for a suspected case (the criteria shown in the supplementary materials) and one of the following pathogenic criteria: 1) a positive real-time PCR-based SARS-CoV-2 nucleic acid test result; 2) high homogeneity between viral gene sequencing results and the known SARS-CoV-2 sequence; and 3) positive serum SARS-CoV-2-specific IgM and IgG antibody test results, or a switch from a negative to a positive SARS-CoV-2-specific IgG antibody test result, or a 4-fold increase in serum SARS-CoV-2-specific IgG antibody levels in the recovery phase relative to the acute phase. In this study, COVID-19 patients who received two sequential negative nucleic acid testing results with sampling interval at least 24 h were classified as having mild or moderate disease according to the baseline severity. Mild cases were those with mild clinical features and no imaging features of pneumonia. Moderate cases were those with fever or respiratory symptoms and imaging features of pneumonia [17].

### Manifestations of COVID-19 on CT imaging

Because mild cases had no imaging features of pneumonia, only patients with moderate COVID-19 underwent chest CT re-examinations. To determine changes in the imaging manifestations during the recovery phase, the chest CT scans were performed at 5 to 7 day intervals over a 2-week period. Typical CT imaging manifestations of COVID-19 include ground-glass density shadows alone, interlobular septal thickening combined with ground-glass changes, intralobular interstitial thickening combined with ground-glass changes, consolidation combined with ground-glass changes, fibrous stripes, solid nodules, and other features such as pleural effusion. All CT images were reviewed by the same doctor (Wang J).

### Statistical analysis

The baseline characteristics were presented for all patients with COVID-19 and for subgroups categorized by the severity of disease (mild or moderate). Data were presented as numbers and frequencies for categorical data or as medians with interquartile ranges (IQRs) for continuous data. The frequencies of categorical variables were compared using the χ^2^ test; Fisher’s exact test was used when the data were limited. The Mann–Whitney test was used to compare median values between two independent groups, while the Wilcoxon test was used to compare two paired groups. All statistical analyses were conducted using the Statistical Package for the Social Sciences (SPSS) version 21.0 software (SPSS Inc., Chicago, IL, USA). A two-sided α of < 0.05 was considered statistically significant.

## Results

### Characteristics of the included participants

Fifty-one patients with confirmed COVID-19 were transferred to our hospital for further treatments after two consecutive negative nucleic acid testing results and were included in this study. The median age was 34.0 years (range: 7 months to 67 years; IQR: 20–44 years). Twenty-four patients (47.1%) were male and 36 (70.6%) had ever traveled to Hubei province (Table 1). The median (IQR) incubation time and time to negative nucleic acid testing were 7.5 (1.0, 11.75) days and 3 (2, 7) days.

**Table 1:** Clinical characteristics of the patients infected with mild-to-moderate SARS-CoV-2

Variables	Total (*n* = 51)	Moderate (*n* = 31)	Mild (*n* = 20)	*P*
Ever went to Hubei (n,%)				0.539
Yes	36 (70.6)	23 (74.2)	13 (65.0)	
No	15 (29.4)	8 (25.8)	7 (35.0)	
Age, years	34.0 (20.0, 44.0)	37.0 (25.0, 48.0)	31.0 (16.0, 37.5)	0.288
Gender (n,%)				0.813
Male	24 (47.1)	15 (48.4)	9 (45.0)	
Female	27 (52.9)	16 (51.6)	11 (55.0)	
Incubation time, days	7.5 (1.0, 11.75)	8.0 (1.75, 12.0)	6.5 (0.0, 9.0)	0.361
Time to negative nucleic acid testing, days	3 (2, 7)	2 (4, 8)	2.5 (1.25, 5.5)	0.291
**Symptoms at admission**				
Fever (n,%)	34 (66.7)	23 (74.2)	11 (55.0)	0.156
Duration of fever, days	5.0 (2.0, 7.0)	5.0 (2.0, 7.5)	4.0 (2.0, 6.0)	0.772
Cough (n,%)	31 (60.8)	22 (71.0)	9 (45.0)	0.083
Expectoration (n,%)	6 (11.8)	4 (12.9)	2 (10.0)	1.000
Sore throat (n,%)	6 (11.8)	3 (9.7)	3 (15.0)	0.668
Headache (n,%)	4 (7.8)	1 (3.2)	3 (15.0)	0.287
Fatigue (n,%)	2 (3.9)	2 (6.5)	0 (0.0)	0.514
Stuffy and runny nose (n,%)	4 (7.8)	1 (3.2)	3 (15.0)	0.287
Gastrointestinal reaction (n,%)	5 (9.8)	4 (12.9)	1 (5.0)	0.636
Chest tightness and dyspnea (n,%)	7 (13.7)	5 (16.1)	2 (10.0)	0.690
**Symptoms after two sequential negative nucleic acid testing**				
Fever (n,%)	0 (0)	0 (0)	0 (0)	–
Cough (n,%)	17 (33.3)	11 (35.5)	6 (30.0)	0.767
Expectoration (n,%)	7 (13.7)	5 (16.1)	2 (10.0)	0.690
Sore throat (n,%)	4 (7.8)	3 (9.7)	1 (5.0)	1.000
Headache (n,%)	2 (3.9)	0 (0.0)	2 (10.0)	0.149
Fatigue (n,%)	3 (5.9)	1 (3.2)	2 (10.0)	0.553
Stuffy and runny nose (n,%)	3 (5.9)	0 (0.0)	3 (15.0)	0.055
Gastrointestinal reaction (n,%)	3 (5.9)	2 (6.5)	1 (5.0)	1.000
Chest tightness and dyspnea (n,%)	3 (5.9)	1 (3.2)	2 (10.0)	0.553
Pulse oximeter O2 saturation, %	97.3 ± 0.95	97.1 ± 0.89	97.6 ± 1.00	0.469
Heart rate, breaths/min	86.7 ± 15.5	85.9 ± 15.7	88.0 ± 15.5	0.938
**Disease history**				
Bronchial or pulmonary disease (n,%)	4 (7.8)	4 (12.9)	0 (0.0)	0.094
Other diseases (n,%)^a^	6 (11.8)	6 (19.6)	0 (0.0)	**0.036**
**Treatment (n,%)**^b^				0.216
Western medicine	17 (34.7)	11 (35.5)	6 (33.3)	
Chinese medicine	15 (30.6)	7 (22.6)	8 (44.4)	
Both	17 (34.8)	13 (41.9)	4 (22.2)	

Chinese medicine: Jin Hua Qing Gan particles, antiviral particles, Xiang Sha Liu Jun pills, etc.

^a^Other underlying diseases include glioma, nephrectomy, hyperlipidemia, gastric tumours, type 2 diabetes and thyroid cancer, etc.

^b^Western medicine: levofloxacin, moxifloxacin, ribavirin, abidol, lopinavir, ritonavir, interferon, thymoxin, chloroquine phosphate, interferon, oseltamivir, etc.

**Fig. 1:**
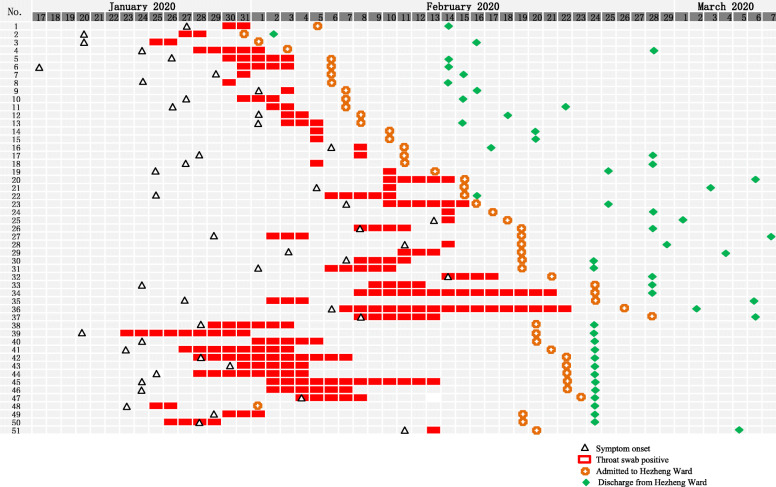
Time course of symptoms, hospital admission after RT-PCR negative, and discharge of patients infected with SARS-CoV-2

Information about the symptom time course, positive throat swab, hospital admission, and discharge for each SARS-CoV-2-infected patient is presented in Fig. 1. Upon hospital admission, 34 of the 51 (66.7%) patients were febrile, with a fever duration of 5.0 (IQR: 2.0, 7.0) days, and 31 of the 51 (60.8%) patients had a cough. Regarding other symptoms, 3.9–13.7% of the patients presented with expectoration, pharyngitis, headache, fatigue, nasal congestion and discharge, gastrointestinal reactions, and chest tightness and dyspnea. Generally, the proportions of symptoms were higher among moderate cases than among mild cases (fever: 74.2% vs. 55.0%; cough: 71.0% vs. 45.0%), but these differences were not statistically significant. None of the patients who were transferred to the Hezheng ward after two consecutive negative nucleic acid tests reported a fever, and the frequency of patients with a cough (17/51, 33.3%) also significantly decreased (both *P* < 0.05). The proportions of the other symptoms generally remained the same. Ten of the 51 (19.6%) patients in the moderate group and none in the mild group had complicating bronchial or lung diseases or other coexisting conditions, such as glioma, nephrectomy, and hyperlipidemia. Regarding treatment, 34.7% of the patients were treated with western medicine only, 30.6% with Chinese medicine only, and 34.8% with both treatments.

### Laboratory parameters

The laboratory results of the included patients with COVID-19 and two consecutive negative nucleic acid tests are presented in Table 2. Compared with the baseline characteristics, a repeated-measures analysis revealed no statistically significant differences 3–5 days after the two consecutive negative nucleic acid tests (Table 3). For longer repeated-measure intervals (> 1 week; Table 4), the laboratory values decreased steadily, but these differences were not statistically significant.

**Table 2:** Laboratory results of the included COVID-19 patients after two sequential negative nucleic acid testing

	N	Total	Moderate		Mild		*P*
			n	Median (IQR)	n	Median (IQR)	
WBC count, × 10^9^ /L	51	5.78 (4.38, 7.70)	31	5.90 (4.84, 8.39)	20	5.62 (3.99, 7.39)	0.298
Neutrophile granulocyte, %	49	55.2 (47.9, 63.3)	30	55.8 (46.9, 63.7)	19	52.4 (41.3, 60)	0.325
Lymphocyte count, ×10^9^ /L	46	2.01 (1.49, 2.53)	28	1.99 (1.41, 2.90)	18	2.07 (1.65, 2.34)	0.839
Lymphocyte, %	51	34.1 (27.9, 41.7)	31	32.3 (23.9, 37.9)	20	35.7 (29.4, 44.6)	0.162
C-reactive protein, mg/L	44	0.73 (0.34, 1.84)	26	0.86 (0.36, 2.04)	18	0.64 (0.21, 1.3)	0.481
D-dimer, μg/mL	20	0.27 (0.22, 0.42)	13	0.25 (0.22, 0.43)	7	0.28 (0.22, 0.39)	0.838
Erythrocyte sedimentation rate, mm/h	10	19 (3.75, 32)	7	19 (4, 24)	3	30 (2, 44)	0.568
Lactate dehydrogenase, U/L	37	362 (323.5, 424)	23	354 (322, 416)	14	369 (322, 448)	0.650
Alanine aminotransferase, U/L	43	21 (16, 36)	27	22 (18, 46)	16	20 (15, 34)	0.606
Aspartate aminotransferase, U/L	43	27 (22, 33)	27	27 (23, 32)	16	26 (20, 33)	0.763

**Table 3:** Laboratory results of the included COVID-19 patients after two sequential negative nucleic acid testing with repeated analyses after 3–5 days

	Total				Moderate				Mild			
	N	After negative	3–5 days after negative	*P*	N	After negative	3–5 days after negative	*P*	N	After negative	3–5 days after negative	*P*
WBC count, ×10^9^ /L	20	7.7 (4.7, 10.2)	6.37 (5.1, 7.65)	0.080	13	7.7 (4.8, 10.2)	6.83 (5.04, 7.74)	0.126	7	7.7 (4.3, 10.4)	6.33 (5.05, 6.92)	0.310
Neutrophile granulocyte, %	18	53.3 (50, 62.5)	52.7 (45.4, 57.6)	0.064	12	55.2 (49, 65.5)	53.3 (43.7, 60.7)	0.099	6	52.8 (45.9, 61.1)	50.2 (43.7, 54.2)	0.463
Lymphocyte count, ×10^9^ /L	15	2.11 (1.65, 2.82)	2.21 (1.66, 2.58)	0.609	10	1.99 (1.52, 2.86)	2.11 (1.51, 2.55)	0.721	5	2.11 (1.9, 4.21)	2.28 (1.76, 3.11)	0.893
Lymphocyte, %	20	34.6 (29.6, 46.1)	34.9 (29.8, 44)	0.904	13	32.3 (26, 39.5)	34.4 (28.5, 42.5)	0.272	7	39.9 (30.5, 52)	35.4 (31.9, 45.1)	0.310
C-reactive protein, mg/L	9	1.92 (1.02, 3.54)	0.87 (0.43, 2.13)	0.123	5	2.35 (1.15, 15.19)	1.24 (0.32, 3.73)	0.500	4	1.2 (0.97, 2.54)	0.75 (0.41, 0.91)	0.109

**Table 4:** Laboratory results of the included COVID-19 patients after two sequential negative nucleic acid testing with repeated analyses after over 1 week

	Total				moderate				Mild			
	N	After negative	1 week after negative	*P*	N	After negative	1 week after negative	*P*	N	After negative	1 week after negative	*P*
WBC count, ×10^9^ /L	15	4.88 (4.16, 8.42)	6.69 (4.6, 7.33)	0.496	10	8.05 (4.84, 10.2)	7.20 (4.60, 7.97)	0.059	5	4.94 (4.07, 5.36)	4.78 (4.22, 6.75)	0.443
Neutrophile granulocyte, %	14	60.1 (46.4, 65.6)	57.2 (49.2, 61.7)	0.802	10	61.3 (56.8, 69.5)	58.1 (51.6, 65.9)	0.114	4	50.2 (47.5, 58.8)	51.9 (47.1, 58.9)	0.844
Lymphocyte count, ×10^9^ /L	12	1.95 (1.42, 2.68)	1.91 (1.38, 2.5)	0.610	8	1.85 (1.42, 2.68)	2.18 (1.24, 2.71)	0.889	4	2.07 (1.43, 2.69)	1.68 (1.52, 2.15)	0.715
Lymphocyte, %	15	30.8 (27.1, 43.4)	30.4 (24.1, 38)	0.363	10	30 (22.1, 32.1)	28.8 (21.8, 35.6)	0.721	5	45.6 (35.4, 50)	33.7 (28.2, 39.3)	0.080
C-reactive protein, mg/L	12	1.23 (0.21, 3.11)	0.43 (0.24, 1.39)	0.131	8	2.14 (0.72, 19.59)	0.86 (0.38, 2.55)	0.128	4	0.16 (0.04, 0.99)	0.28 (0.11, 0.47)	1.000

### CT imaging manifestations

**Fig. 2:**
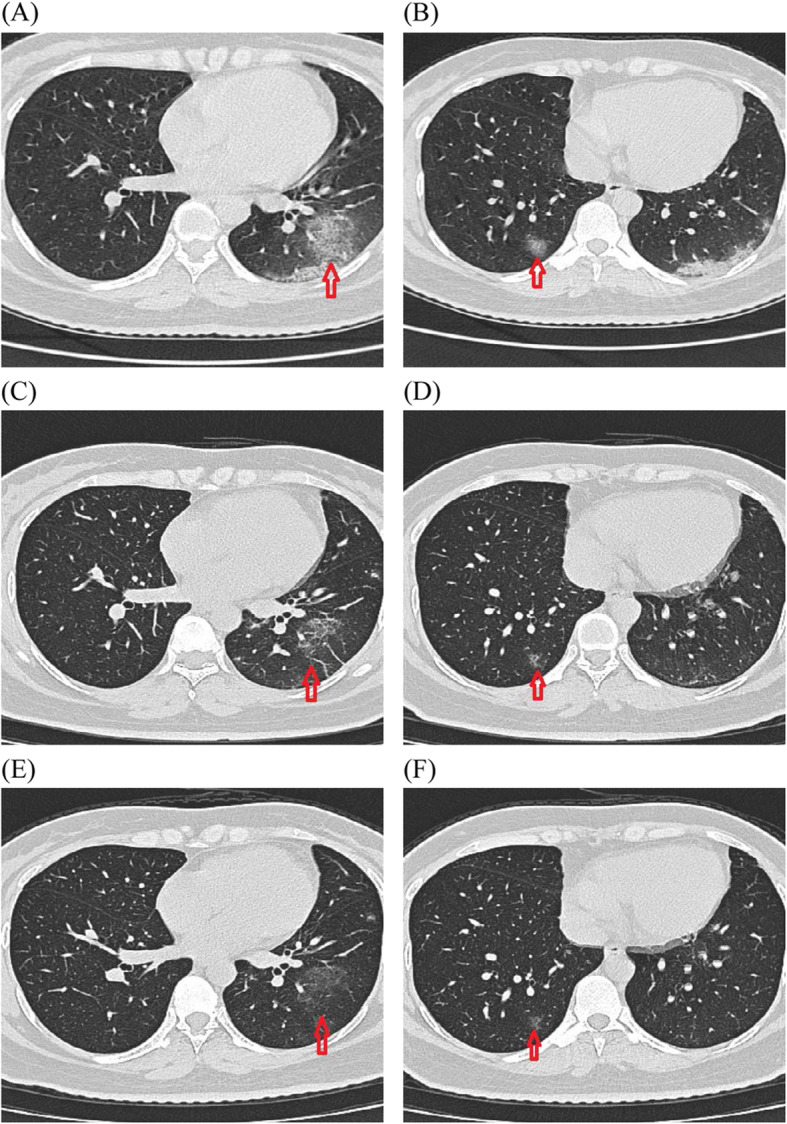
Transverse chest computed tomograms from a patient, showing ground glass opacity and consolidation of lower lobe of right and left lung on day 1 after negative for SARS-CoV-2 (**a** and **b**), on day 10 after negative for SARS-CoV-2 with absorption of 60–70% (**c** and **d**), and on day 16 after negative for SARS-CoV-2 with absorption of 80–90% (**e** and **f**)

All patients with moderate COVID-19 had undergone a chest CT scan by the time of transfer to the Hezheng ward. Among these patients (Table 5 and Fig. 2), only one lobe was affected in 7 (22.6%) patients and two or more lobes were affected in 24 (77.4%). The left lower lobe was the most common location of lesions (71.0%, 22/31), followed by the right lower lobe (61.3%, 19/31), left upper lobe (48.4%, 15/29), and right middle lobe (38.7%, 12/29). Right upper and lower and left lower lobe involvement were generally distributed evenly between cases with single affected lobe and those with multiple affected lobes. However, 83.3% (10/12) of the cases with right middle lobe involvement only involved a single lobe and 66.7% (10/15) of the cases with left upper lobe involvement had multiple affected lobes.

**Table 5:** Lung lobes involved of 31 moderate COVID-19 after two sequential negative nucleic acid testing

	Total	Single	Multiple
**Number of lobes involved**			
1	7 (22.6)	–	–
2	12 (38.7)	–	–
3	5 (16.1)	–	–
4	4 (12.9)	–	–
5	3 (9.7)	–	–
More than two lobes involved	24 (77.4)	–	–
**Lobe of lesion distribution**			
Right upper lobe	9 (31.0)	5 (55.6)	4 (44.4)
Right middle lobe	12 (41.4)	10 (83.3)	2 (16.7)
Right lower lobe	19 (65.5)	9 (47.3)	10 (52.6)
Left upper lobe	15 (51.7)	5 (33.3)	10 (66.7)
Left lower lobe	22 (75.9)	11 (50.0)	11 (50.0)

The most common CT imaging manifestations were ground-glass opacities (51.6%, 16/31) and fibrous stripes (54.8%, 17/31) (Table 6 and Supplementary Table 1). Twelve of the 31 patients underwent repeated chest CT scans after negative nucleic acid test results; of them, seven patients (58.3%) exhibited a > 60% reduction in the ground-glass opacities. However, a reduction < 5% was observed in four patients. In case 17, the ground-glass opacity with a reduction of < 5% persisted for almost month. In addition, the 12 patients with negative throat swabs (case nos. 38–47, 49, and 50) occasionally had repeated positive fecal tests (Table 7 and Supplementary Table 2).

**Table 6:** CT imaging of 31 moderate COVID-19 after two sequential negative nucleic acid testing

	Moderate (*n* = 31)
**Density and inner features**	
Ground glass opacity	16 (51.6)
Thickened intralobular septa	7 (22.6)
Thickened interlobular septa	3 (9.7)
Mixed ground glass opacity and consolidation	3 (9.7)
Fibrous stripes	17 (54.8)
Consolidation	8 (25.8)
**Other features**	
Pleural effusion	3 (9.7)

**Table 7:** Percentage change of CT ground-glass absorption of 12 cases after two sequential negative nucleic acid testing

	After 1 week	After 2 week	After 3 week	After 4 week
< 5%	2	3	1	1
5 ~ 50%	1	0	0	0
51 ~ 70%	2	0	1	0
70 ~ 80%	3	0	0	0
> 80%	2	4	1	1

## Discussion

This descriptive case series evaluated the characteristics of 51 patients with COVID-19 who had obtained two consecutive negative nucleic acid testing results, and we present herein the clinical and CT imaging features of these cases. Generally, the patients’ clinical data continued to return to normal and nearly half of the patients with moderate COVID-19 who underwent repeated chest CT scans after two consecutive negative nucleic acid test results exhibited a > 60% reduction in the ground-glass opacities on CT within 1 week, suggesting that further attention should be needed. In addition, we determined that positive fecal SARS-CoV-2 tests were common in these patients.

Recently, a study showed that, even after suffering twice consecutive negative nucleic acid tests, residual pulmonary lesions including ground-glass opacities, pulmonary interstitium thickening, and pleural effusion were still visible in CT images, particularly among the severe/critical cases [15]. Although this result was consistent with our finding, there was not regular interval time between negative nucleic test and CT re-examinations in their study, and they failed to explore the changes in follow-up CT findings of patients, as well as the situations in those with re-positive RT-PCR tests.

Since the first occurrence of COVID-19 in Wuhan, China, in December 2019 [18, 19], this disease has spread rapidly across the world. The rapid increase in the number of COVID-19 cases has led to an insufficiency of health-care resources [20]. In China, the government issued policies and criteria for the diagnosis and treatment of COVID-19 with the goal of improving the availability and accessibility of health care resources in China. In Shenzhen, China, all patients with suspected and confirmed COVID-19 were sent to a government-assigned hospital for diagnosis and initial treatment, and those who met the discharge criteria but continued to exhibit clinical symptoms were transferred to other hospitals for further treatment. This strategy helped to improve the quality and utilization of medical services and resources. However, the effectiveness and usefulness of this further treatment remain unclear.

In our study, fever and cough were the most common symptoms at diagnosis, consistent with the findings from a meta-analysis of 61 studies (59,254 patients) [12] that identified fever [82, 95% confidence interval (CI): 56–99%] and cough (61, 95% CI: 39–81%) as the most common disease-related symptoms. We found that patients with moderate COVID-19 were more likely to have comorbidities than those with mild disease. In our sample, no patient presented with fever after receiving negative nucleic acid tests and the proportion of patients with cough also decreased significantly (60.8% vs. 33.3%), indicating that the initial symptomatic treatments were effective. However, the proportions of other symptoms, such as pharyngitis, nasal congestion and discharge, and dyspnea, generally remained unchanged, but the non-specific symptoms were observed.

In our analysis of CT data, the left lower lobe was the most affected site, followed by the right lower lobe. This finding is consistent with the findings of previous radiological studies of patients with COVID-19 [21, 22]. The lower lobes are commonly affected because of the anatomical structure of the trachea and bronchi; i.e., the right bronchus is short and straight. By the time the patients in our study were transferred to the Hezheng ward, the most common CT imaging manifestations were ground-glass opacities alone and strip-like density shadows. We found that serial CT imaging enabled the continuous monitoring of disease changes in our patients.

At the end of the first week, the lesion extent decreased by > 50% relative to the baseline in 7 of 12 patients with repeated CT scan data. However, half of the patients continued to manifest abnormal imaging findings, and four cases exhibited a < 5% reduction in lung lesions within 1 week. This phenomenon suggests that the virus might remain in the human body at a level below the detection threshold level of the throat swab test, which would lead to a negative result [23], and is consistent with previous finding that chest CT was more sensitive than RT-PCR [24]. Some patients with negative throat swab test results also received repeated positive fecal test results, which suggested incomplete elimination of the virus and a continued risk of transmission. Although the participants’ clinical features continued to resolve after receiving negative nuclear acids test results and treatment, their imaging features remained abnormal. Our results and those of other studies indicate that further isolation and treatment is necessary even after a negative throat swab nuclear acid test if clinical symptoms, epidemiological characteristics, and chest CT imaging characteristics of viral pneumonia compatible with COVID-19 infection are present [25]. We note, however, that one patient (case 17) exhibited a decrease in the ground-glass opacity of < 5% over a period of almost 1 month. As this patient was asymptomatic and consistently received negative nuclear acid test results, we speculate that the abnormal imaging findings might indicate old lesions caused by previous respiratory diseases.

This study had several limitations. First, the COVID-19 cases were confirmed through nucleic acid tests of respiratory tract specimens. After a patient received two consecutive negative respiratory tract sample-based test results, some of them were transferred to our hospital for further treatment until their clinical symptoms had mostly improved. We note that some patients continued to yield positive fecal tests. However, the clinical characteristics and dynamic imaging changes in these cases during hospitalization could supplement the current evidence on COVID-19. Second, given the small sample size, it was difficult to systematically explore the differences between mild and moderate cases, and the risk of a type I error should be considered. Moreover, because the analyses were not adjusted for multiple comparisons, the findings should be interpreted as exploratory and descriptive. Third, the 51 patients in this study had different requirements and received various treatments, including drug therapies and traditional Chinese medicine, which may be an important confounder in data comparisons. Finally, as this was a retrospective study, some data could not be collected or measured. For example, less than half of the patients had data from repeated clinical measurements.

## Conclusions

In this retrospective cohort study, we found that the clinical features and abnormal CT scanning appearance could resolve after twice consecutive negative nucleic acid test. However, patients who manifested abnormal imaging features should maintain further isolation and treatment to monitor further transmission of COVID-19. More studies with larger sample size are needed to further explore the clinical and imaging features in patients with COVID-19, to improve evaluation of remission and to help clinicians to make decision.

## Supplementary Information

**Additional file 1: Supplementary Table 1.** Percentage change of CT ground-glass absorption after two sequential negative nucleic acid testing. **Supplementary Table 2.** Patients with repeat focal positive tests.

**Additional file 2: Appendix A.** Criteria for epidemiological history and suspected case of COVID-19.

## Data Availability

The data analyzed during this study are available from the corresponding author on reasonable request.

## Abbreviations

COVID-19: Coronavirus disease 2019
CT: Computed tomography
IQR: Interquartile range
PHEIC: Public Health Emergency of International Concern
RT-PCR: Reverse-transcription-polymerase chain-reaction
SARS-CoV-2: Severe acute respiratory syndrome coronavirus 2
SPSS: Statistical Package for the Social Sciences
WHO: World Health Organization
