# Supplementary material for: Clinical and CT features of mild-to-moderate COVID-19 cases after two sequential negative nucleic acid testing results: a retrospective analysis
Source: BMC Infect Dis. 2021 Apr 8;21:333. doi: 10.1186/s12879-021-06013-x (PMC8027977; doi:10.1186/s12879-021-06013-x)
Supplement: Supplementary file 1 — Additional file 1: Supplementary Table 1. Percentage change of CT ground-glass absorption after two sequential negative nucleic acid testing. Supplementary Table 2. Patients with repeat focal positive tests. [file 12879_2021_6013_MOESM1_ESM.docx]

**Supplementary table 1** Percentage change of CT ground-glass absorption after two sequential negative nucleic acid testing

| Patients No. | Date of examination | Time lag after two sequential negative nucleic acid testing | Percentage change of CT ground-glass absorption |
| --- | --- | --- | --- |
| 3 | Feb. 5 | 0 | Baseline |
|  | Feb. 12 | 7 | 70%-80% |
| 9 | Feb. 3 | 0 | Baseline |
|  | Feb. 8 | 5 | 90-100% |
|  | Feb. 14 | 11 | 100% |
| 11 | Feb. 8 | 0 | Baseline |
|  | Feb. 13 | 5 | 70-80% |
|  | Feb. 20 | 12 | 80-90% |
|  | Mar. 7 | 27 | 100% |
| 13 | Feb. 4 | 0 | Baseline |
|  | Feb. 13 | 9 | ＜5% |
| 14 | Feb. 13 | 0 | Baseline |
|  | Feb. 18 | 5 | 70%-80% |
| 17 | Feb. 14 | 0 | Baseline |
|  | Feb. 26 | 12 | ＜5% |
|  | Mar. 5 | 19 | ＜5% |
|  | Mar. 13 | 27 | ＜5% |
| 19 | Feb. 14 | 0 | Baseline |
|  | Feb. 20 | 6 | ＜5% |
| 21 | Feb. 16 | 0 | Baseline |
|  | Feb. 22 | 6 | 60-70% |
|  | Feb. 28 | 12 | 80-90% |
| 23 | Feb. 16 | 0 | Baseline |
|  | Feb. 23 | 7 | ＞90% |
| 27 | Feb. 20 | 0 | Baseline |
|  | Feb. 28 | 8 | 60-70% |
|  | Mar. 5 | 13 | 80-90% |
| 29 | Feb. 20 | 0 | Baseline |
|  | Feb. 28 | 8 | ＜5% |
| 35 | Feb. 1 | 0 | Baseline |
|  | Feb. 3 | 2 | 30-40% |
|  | Feb. 25 | 24 | 60-70% |
|  | Mar. 4 | 31 | ＞90% |

**Supplementary table 2** Patients with repeat focal positive tests

| Patients No. | Date with focal test | Focal results | Throat swab results |
| --- | --- | --- | --- |
| 38 | Feb. 18 | Positive | Negative |
| 39 | Feb. 18 | Positive | Negative |
| 40 | Feb. 19 | Positive | Negative |
| 41 | Feb. 21 | Positive | Negative |
| 42 | Feb. 21 | Positive | Negative |
| 43 | Feb. 21 | Positive | Negative |
| 44 | Feb. 21 | Positive | Negative |
| 45 | Feb. 21 | Positive | Negative |
| 46 | Feb. 20 | Positive | Negative |
| 47 | Feb. 22 | Positive | Negative |
| 49 | Feb. 20 | Positive | Negative |
| 50 | Feb. 18 | Positive | Negative |
