# Supplementary material for: Clinical and CT features of mild-to-moderate COVID-19 cases after two sequential negative nucleic acid testing results: a retrospective analysis
Source: BMC Infect Dis. 2021 Apr 8;21:333. doi: 10.1186/s12879-021-06013-x (PMC8027977; doi:10.1186/s12879-021-06013-x)
Supplement: Supplementary file 2 — Additional file 2: Appendix A. Criteria for epidemiological history and suspected case of COVID-19. [file 12879_2021_6013_MOESM2_ESM.docx]

**Appendix A** Criteria for epidemiological history and suspected case of COVID-19

| No. | Criteria | Note |
| --- | --- | --- |
| **Epidemiological history** | | |
| 1) | travel to or residence in Wuhan or other cities with continuous local SARS-CoV-2 transmission in the 14 days before symptom onset; |  |
| 2) | contact with SARS-CoV-2-infected patients in the 14 days before symptom onset; |  |
| 3) | contact with patients with a fever or respiratory symptoms from Wuhan or other cities with continuous local transmission in the 14 days before symptom onset; |  |
| 4) | clustered onsets. |  |
| **Definition of suspected cases** | | |
| 1) | fever and/or respiratory symptoms; | 1) defined as a patient with one epidemiological history and any two clinical features of COVID-19;  2) without an epidemiological history but with all of the clinical features. |
| 2) | imaging features consistent with COVID-19; |  |
| 3) | a normal or reduced total white blood cell count and/or normal or reduced lymphocyte count during the early stages of disease. |  |
